# Lupus and Kikuchi-Fujimoto Disease: A Combination for Catastrophe

**DOI:** 10.7759/cureus.27423

**Published:** 2022-07-28

**Authors:** Subramani Jagadeesan, Shilpi Rani, Yogesh C Porwal, Pranav Patel

**Affiliations:** 1 Internal Medicine, Vardhman Mahavir Medical College and Safdarjung Hospital, New Delhi, IND; 2 Internal Medicine, Sir Ganga Ram City Hospital, New Delhi, IND

**Keywords:** bull neck, kfd with diffuse lymphadenopathy, lymphadenopathy, systemic lupus erythematosus, kikuchi-fujimoto disease and systemic lupus erythematosus

## Abstract

Kikuchi-Fujimoto disease (KFD) is a self-limiting disease of idiopathic origin affecting young women characterized by unexplained fever and lymphadenopathy. It has been usually found to be associated with autoimmunity, of which systemic lupus erythematosus (SLE) is the most outstanding. Fever and lymphadenopathy carry a broad differential, and a missed diagnosis of a rare condition such as Kikuchi can lead to inappropriate treatment in an otherwise benign condition. Therefore, careful examination and histologic confirmation of the diagnosis are critical.

## Introduction

Kikuchi disease, also called Kikuchi-Fujimoto disease (KFD) or Kikuchi histiocytic necrotizing lymphadenitis, is a benign self-limiting condition of unknown etiology, characterized typically by unexplained febrile illness and cervical lymphadenopathy. KFD has a global distribution, with a relatively higher prevalence in the Asian and Japanese populations [1]. Alike other autoimmune ailments, KFD stands out in females (4:1) with the median age of presentation being 30 years. The etiology of KFD is still debated in the literature; however, its clinical presentation, course, and histologic changes suggest T-cell and histiocyte response to an infectious agent. Patients with KFD can mimic lymphoma, viral infections, and other autoimmune conditions; thus, it becomes imperative to diagnose KFD to avoid needless treatment [2]. Herewith, we report a case of KFD that copresented with florid signs of systemic lupus erythematosus (SLE) in a 27-year-old female.

## Case presentation

**Figure 1:**
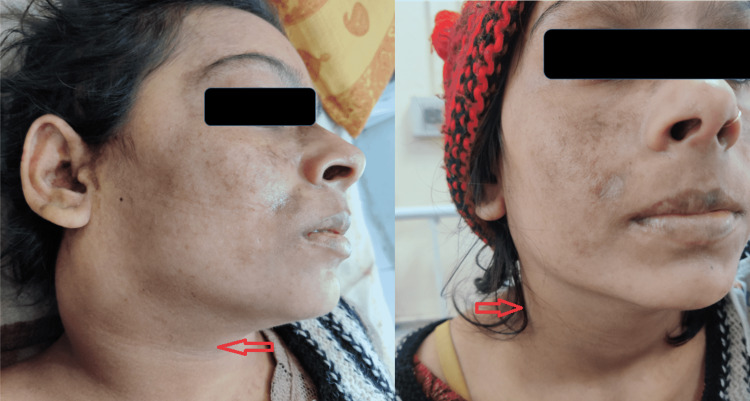
Comparison of physical findings before and after treatment Characteristic malar rash and “bull neck” appearance on the left image with the resolution of the latter posttreatment on the right image (arrows).

A 27-year-old female presented with acute-onset high-grade fever for 14 days associated with easy fatiguability and bilateral painful swallowing. She had characteristic malar rash sparing nasolabial folds (Figure 1, left) and erythematous, pruritic maculopapular rash on bilateral hands for the past 10-12 days. She also complained of severe polyarthralgia, hair loss, and painful oral ulcers for the past 15 days. The patient had decreased appetite and undocumented weight loss over the past quarter alongside irregular menstrual cycles. She denies any recreative substance use nor suffers from any medical illness a priori. Neither was there a significant history of connective tissue diseases in her family. On clinical assessment, she had conjunctival pallor and bilateral cervical lymph node enlargement, with the largest dimension of approximately 5 cm. The nodes were nodular, firm, non-matted, and warm on touch with minimal tenderness. Non-scarring alopecia and multiple erythematous, maculopapular, non-tender, non-blanching rashes were noted in bilateral hands along with butterfly malar rash over the cheeks. No signs of synovitis could be elicited, although hepatomegaly of 17 cm was evident. The laboratory findings are summarized in Table 1.

**Table 1:** Initial laboratory findings TLC: total leukocyte count; DLC: differential count; ESR: erythrocyte sedimentation rate; MCV: mean corpuscular volume; MCH: mean corpuscular hemoglobin; AST: aspartate aminotransferase; ALT: alanine aminotransferase; ALP: alkaline phosphatase; CRP: C-reactive protein; LDH: lactate dehydrogenase; SGOT: serum glutamic oxaloacetic transaminase; SGPT: serum glutamic pyruvic transaminase

Laboratory parameters	Patient’s value	Normal range
Hemoglobin (g/dL)	7.6	11-15
TLC (cells/mm^3^)	4,000	4-11
Platelets (L/mm^3^)	1.7	1.5-4.5
DLC	N72/L22	
ESR (mm/hour)	43	<15
Serum procalcitonin (ng/mL)	1.80	<0.1
MCV (fL)	83	80-100
MCH (pg)	28	27-31
Total bilirubin (mg/dL)	0.6	<1.2
SGOT (U/L)	250	8-48
SGPT (U/L)	124	7-55
ALP (U/L)	117	45-147
CRP (mg/dL)	3.2	<0.3
LDH (U/L)	413	230-460
Serum ferritin (mcg/L)	1,575	25-336

The investigation revealed bicytopenia (anemia and leukopenia), with raised inflammatory markers and transaminitis. Urine examination R/M showed 2+ moderately increased proteinuria and hematuria.

**Figure 2:**
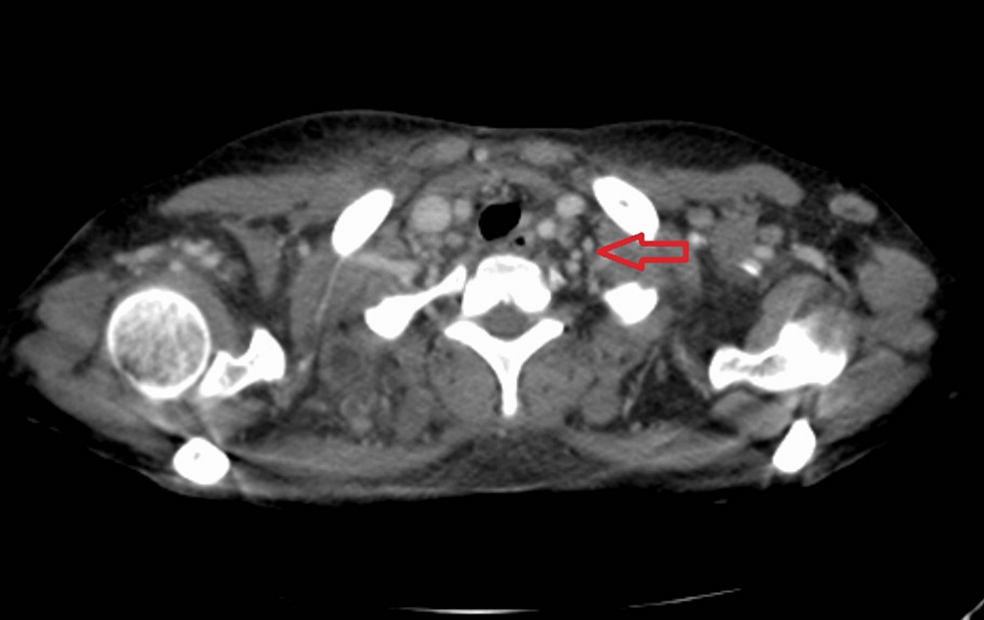
CECT of the neck depicting numerous cervical and axillary adenopathy Multiple discrete non-matted inflamed lymph nodes are evident at the level of the posterior triangle of the neck and anterior axilla (arrow).

**Figure 3:**
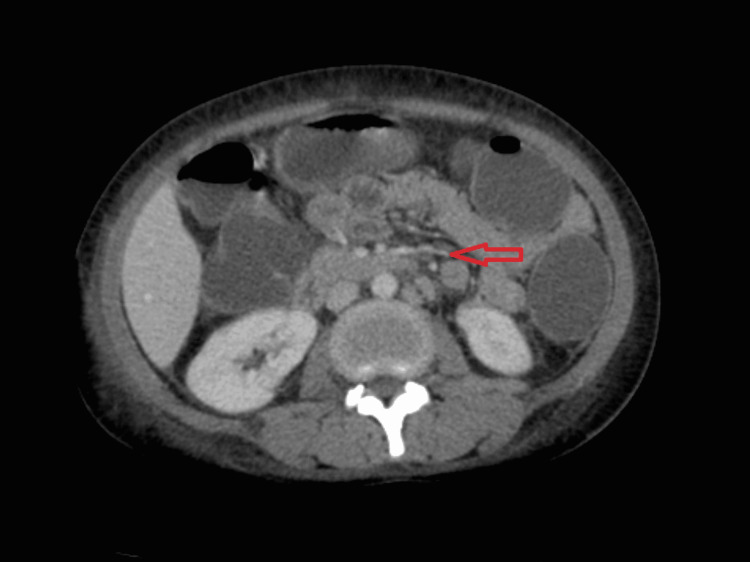
CECT of the abdomen revealing para-aortic and mesenteric adenopathy

A basic workup for fever and causes of generalized lymphadenopathy was done. Local and possible tropical infections were ruled out. Blood and urine cultures were sterile. Polymerase chain reaction (PCR) for Epstein-Barr virus (EBV) and cytomegalovirus (CMV) were negative. Histoplasma urine antigen was found to be negative. Ultrasonogram of the neck was suggestive of enlarged necrotic lymph nodes in the right parotid, posterior triangle, level 2-4, with the largest measuring 1.9 × 4.8 cm, and right submandibular sialadenitis. PCR of lymph node aspirate was negative for *Mycobacterium tuberculosis*. Contrast-enhanced CT (CECT) of the neck, thorax, and abdomen was suggestive of multiple heterogeneously enhancing necrotic bilateral cervical and axillary lymph nodes alongside serous cavity effusions (Figures 2, 3).

**Figure 4:**
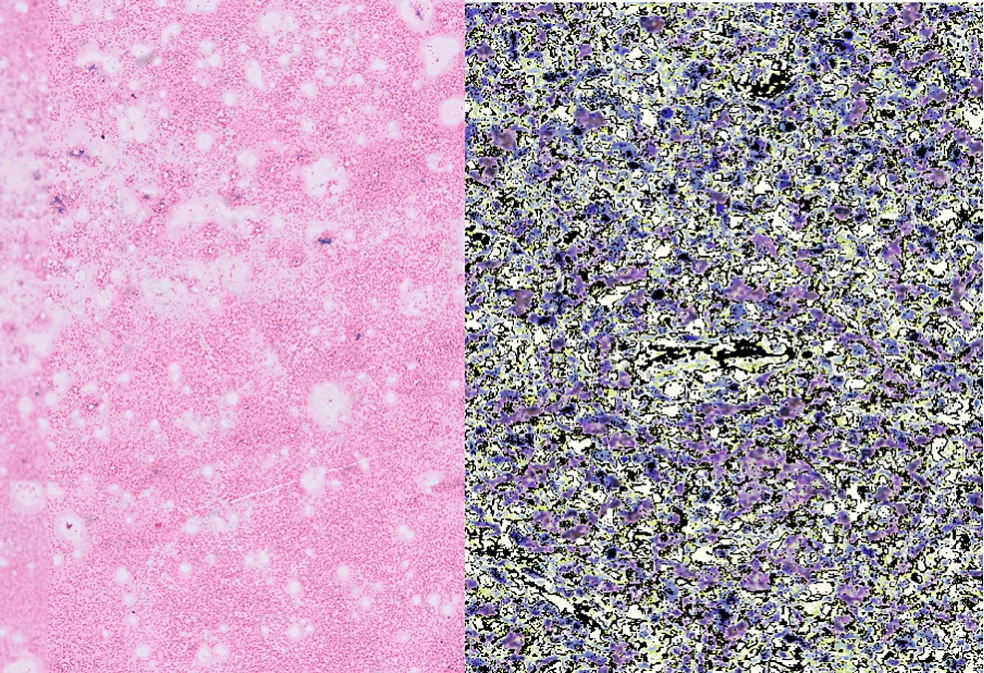
FNAC of the node illustrating (left) proliferation, intercurrent necrosis, and numerous apoptotic cells, and IHC demonstrating CD68-reactive histiocytes

The possibilities of acute HIV syndrome, secondary syphilis, infectious mononucleosis, Hodgkin’s lymphoma, drug rash with eosinophilia and systemic symptoms (DRESS) syndrome, and SLE were considered, with ulceroglandular tularemia, drug eruptions, adult-onset Still’s disease, dermatomyositis, and other atypical tropical bacterial infections such as rickettsiosis as relatively rare possibilities. The rest being ruled out, the serum antinuclear antibody (ANA) of the patient was positive with a homogeneous pattern and titer of 1:640. Anti-double-stranded DNA (ds-DNA), anti-Smith, and anti-ribosomal P antibodies were found to be positive too. No atypical cells were seen on the peripheral blood smear. Bone marrow aspirate showed cellular reactive bone marrow with a normal M:E ratio. Bone marrow biopsy revealed overall cellularity of 75%-80% with no lymphoid aggregates. Lymph node biopsy showed focal necrosis in cortical and paracortical areas with marked karyorrhexis and proliferation of distinctive crescentic histiocytes and plasmacytoid monocytes, which was characteristic of KFD, with an absence of neutrophils and plasma cells. Immunohistochemical staining showed CD68-positive histocytes and CD8-positive T-cells (Figure 4).

The patient was managed with aggressive glucocorticoid therapy along with hydroxychloroquine (HCQ). She responded well to treatment with resolution of symptoms (Figure 1), and the remaining hospital course was uneventful. Outpatient review after three months revealed that the patient’s inflammatory markers, viz., quantitative hs-CRP, complement levels, and anti-ds-DNA levels, were in a falling trend. Her anemia improved and was at the level of 10.4 g/dL then. She is still under follow-up with no recurrence of the disease to date.

## Discussion

Kikuchi disease, which was discovered initially in Japan in 1972, is considered a disease in young women with the median age of presentation around 30 years. Early reports suggested a female/male ratio of 4:1; however, recent reports seem to indicate that the female preponderance was overemphasized in the past and that the actual ratio is much closer to 1:1 [1,3]. There is much speculation about the etiology of the disease, although the exact pathogenic mechanism is yet to be discovered. The typical clinical presentation and histologic changes do suggest an immunologic response of T-cells and histiocytes to usually an infectious agent including, but not limited to, EBV, human herpesviruses (HHV 6 and 8), human immunodeficiency virus (HIV), *Yersinia enterocolitica*, and *Toxoplasma* spp. KFD shares sex and age predisposition, as well as histologic features, with systemic lupus erythematosus (SLE), and KFD precedes lupus in most of the longitudinal studies, although it is a copresentation in this patient [2].

Conventionally, with a subacute presentation, lymphadenopathy is a constant finding, especially in the cervical band, although other groups could also be involved. Fever is also a primary symptom in around 30%-50% of patients, thus making it a common differential diagnosis for chronic febrile illness as in connective tissue diseases in the Western countries and infections, viz., tuberculosis and brucellosis, in the tropics [2,4]. Rash, polyarthritis, fatigue, hepatosplenomegaly, and a few sporadic symptoms such as rigors, myalgia, arthralgia, night sweats, diarrhea, and weight loss appear to be more prominent in patients with extra-nodal disease.

KFD is known to occur in conjunction with SLE. In 30% of cases, it was seen that Kikuchi presents before SLE, and in 23% of cases, Kikuchi was found to follow SLE, although, in clinical practice, the former appears to be a commoner occurrence [5]. KFD, although a diagnosis of exclusion, is generally diagnosed based on an excisional biopsy of affected lymph nodes. Leukopenia, transaminitis, and raised serum lactate dehydrogenase (LDH) may sometimes hint at the diagnosis. Macrophage activation syndrome was described in 30% of a series of hospitalized patients, associated with longer hospital stays and increased late glucocorticoid use. The characteristic histopathologic findings of KFD include irregular paracortical areas of coagulative necrosis with abundant karyorrhectic debris, which can distort the nodal architecture, and many different types of histiocytes at the margin of the necrotic areas, with immunophenotyping suggestive of predominant T-cells [1,4,6].

Although a self-limiting illness lasting for months with simple and conservative measures, there is no proven effective treatment for KFD as of now. Symptomatic measures aimed to relieve distressing local and systemic complaints with analgesics, antipyretics, and NSAIDs may be used to alleviate lymph node tenderness and fever if coexistent. Patients with severe or persistent symptoms may be managed with glucocorticoids with intravenous immunoglobulins, but not always with apparent benefit in a few prior research [2,6,7].

## Conclusions

Kikuchi disease is an uncommon, self-limiting disease with an excellent prognosis when identified and managed appropriately. Not always does KFD precedes SLE as frequently reported in the literature; sometimes, they copresent, leading to perplexity in arriving at an early diagnosis. Awareness of the disease among clinicians is exceedingly important, especially when a patient with fever and lymphadenopathy is assessed to prevent any misdiagnosis and inappropriate treatment as most of them tend to get diagnosed erroneously as either lymphoma or even tuberculosis sometimes. Although most of them resolve spontaneously with supportive care, recurrent and severe necrotizing lymphadenitis is increasingly managed with hydroxychloroquine (HCQ), corticosteroids, and anakinra (recombinant human interleukin-1 receptor antagonist). Long-term follow-up is substantial to survey the possibility of relapse and the possible development of SLE or other lymphoproliferative malignant diseases later in life.
